# Silvestrol induces early autophagy and apoptosis in human melanoma cells

**DOI:** 10.1186/s12885-015-1988-0

**Published:** 2016-01-13

**Authors:** Wei-Lun Chen, Li Pan, A. Douglas Kinghorn, Steven M. Swanson, Joanna E. Burdette

**Affiliations:** Department of Medicinal Chemistry and Pharmacognosy, University of Illinois at Chicago, Chicago, IL 60607 USA; Division of Medicinal Chemistry and Pharmacognosy, College of Pharmacy, Ohio State University, Columbus, OH 43210 USA; School of Pharmacy, University of Wisconsin-Madison, Madison, WI 53705 USA

**Keywords:** Melanoma, Silvestrol, Autophagy, Apoptosis

## Abstract

**Background:**

Silvestrol is a cyclopenta[*b*]benzofuran that was isolated from the fruits and twigs of *Aglaia foveolata*, a plant indigenous to Borneo in Southeast Asia. The purpose of the current study was to determine if inhibition of protein synthesis caused by silvestrol triggers autophagy and apoptosis in cultured human cancer cells derived from solid tumors.

**Methods:**

In vitro cell viability, flow cytometry, fluorescence microscopy, qPCR and immunoblot was used to study the mechanism of action of silvestrol in MDA-MB-435 melanoma cells.

**Results:**

By 24 h, a decrease in cyclin B and cyclin D expression was observed in silvestrol-treated cells relative to control. In addition, silvestrol blocked progression through the cell cycle at the G_2_-phase. In silvestrol-treated cells, DAPI staining of nuclear chromatin displayed nucleosomal fragments. Annexin V staining demonstrated an increase in apoptotic cells after silvestrol treatment. Silvestrol induced caspase-3 activation and apoptotic cell death in a time- and dose-dependent manner. Furthermore, both silvestrol and SAHA enhanced autophagosome formation in MDA-MB-435 cells. MDA-MB-435 cells responded to silvestrol treatment with accumulation of LC3-II and time-dependent p62 degradation. Bafilomycin A, an autophagy inhibitor, resulted in the accumulation of LC3 in cells treated with silvestrol. Silvestrol-mediated cell death was attenuated in ATG7-null mouse embryonic fibroblasts (MEFs) lacking a functional autophagy protein.

**Conclusions:**

Silvestrol potently inhibits cell growth and induces cell death in human melanoma cells through induction of early autophagy and caspase-mediated apoptosis. Silvestrol represents a natural product scaffold that exhibits potent cytotoxic activity and could be used for the further study of autophagy and its relationship to apoptosis in cancer cells.

**Electronic supplementary material:**

The online version of this article (doi:10.1186/s12885-015-1988-0) contains supplementary material, which is available to authorized users.

## Background

Skin cancer is the most commonly diagnosed cancer. Melanoma accounts for less than two percent of skin cancers, but approximately 75 % of skin cancer deaths are a result of melanoma [1]. Melanoma is often considered one of the most aggressive and treatment-resistant human cancers. Discoveries have shown that melanoma frequently harbors mutations that attenuate tumor suppressor genes such as p53 and PTEN, leading to cell cycle dysregulation. Melanoma also frequently exhibits enhanced activation of receptor tyrosine kinases like epidermal growth factor receptor (EGFR) and MET, as well as BRAF and small G proteins such as Ras [2]. Together, these aberrant signaling networks render melanoma resistant to conventional chemotherapeutic drugs.

For decades, secondary metabolites from plants, fungi and bacteria have been found to exhibit potent anticancer activity [3–6]. The genus *Aglaia* of the plant family Meliaceae consists of over 100 species of dioecious trees or shrubs with small fragrant flowers indigenous to the tropical rain forests of Indonesia and Malaysia, as well as other southeast Asian countries. Previous phytochemical studies on *Aglaia* species have shown that among all the isolates, cyclopenta[*b*]benzofurans, also known as rocaglate or rocaglamide derivatives, deserve further study due to their unusual carbon skeletons [7, 8] and potent biological activities. Silvestrol is a rocaglate derivative containing a dioxanyl ring and was isolated from the tropical tree *Aglaia foveolata.* Silvestrol is toxic against human cancer cell lines propagated in vitro or in vivo with a potency similar to that of paclitaxel or camptothecin [9]. It is a translation initiation inhibitor that prevents ribosome loading onto a mRNA template by targeting the eukaryotic initiation factor, eIF-4A [10, 11]. Silvestrol was found to possess potent anticancer activities in both the in vivo hollow fiber assay and the P-388 lymphocytic leukemia mouse model [9]. The compound has been found to show promising in vitro and in vivo activities against certain B-cell malignancies [12], and has been under preclinical toxicogical development in the National Cancer Institute Experimental Therapeutics (NExT) program. However, the mechanism of action of silvestrol responsible for inducing cellular death is still unclear. Tight control of protein synthesis is essential for normal cellular function and survival, but unrestrained protein synthesis can promote tumorigenesis. Therefore, silvestrol’s ability to block protein synthesis is of significant interest in potentially treating cancers.

Autophagy is an essential, homeostatic process involving the lysosomal degradation of cytoplasmic organelles or cytosolic components. Autophagy is a physiological process involved in the routine turnover of proteins or intracellular organelles [13]. The process of autophagy starts by sequestering cytosolic proteins or organelles into autophagosomes that then fuse with lysosomes to form autolysosomes for the degradation of sequestered contents by lysosomal hydrolases [14]. Control of autophagy relies on proteins encoded by a set of autophagy-related genes [15]. First, autophagosome nucleation is mediated by Beclin 1 (Atg6), a class III phosphatidylinositol 3-kinase complex [16, 17]. Later, the Atg12-Atg5 complex and microtubule-associated protein 1 light chain 3 (LC3, Atg8) are required for the elongation of autophagosomes. During autophagy, LC3-II is increased from the conversion of LC3-I, which is considered an autophagosomal marker [18]. Autophagy may protect against cancer by promoting autophagic cell death or contribute to cancer cell survival. Importantly, autophagy and apoptosis often occur in the same cell, mostly in a sequence in which autophagy precedes apoptosis. Loss or gain of either autophagy or apoptosis influences numerous pathological processes [19, 20]. Proteins involved in pathways that modify autophagy might provide novel anticancer targets [21, 22].

Tight regulation of protein synthesis is critical for cell survival during nutrient and growth factor deprivation. In the presence of adequate nutrients, protein synthesis is stimulated and autophagy is inhibited [23, 24]. Tumor growth requires new protein synthesis. Therefore, use of silvestrol that inhibits translation could be a useful therapeutic strategy [25]. Oncogenic effects arising from the ectopic expression of the eukaryotic initiation factor eIF-4E has been reported [25]. Moreover, down-regulation of eIF-4E, which is the rate-limiting factor for translation, has been shown to have an anti-tumor effect [26]. Considerable attention has therefore been focused on targeting other components of the protein translation machinery. As a translation inhibitor with a unique structure, silvestrol previously showed histological selectivity for several cancer cell types, perhaps through the depletion of short half-life pro-growth or pro-survival proteins, including cyclin D and Mcl-1. Given its ability to modulate tumor cell growth, the current study evaluates whether silvestrol induces both apoptosis and autophagy to induce cell death, and further defines the mechanism of this agent.

## Methods

### Reagents and antibodies

The isolation of silvestrol, {6-*O*-demethyl-6-[6-(1,2-dihydroxyethyl)-3-methoxy-1,4-dioxan-2-yl]-aglafolin}, has been described previously [9], and this compound was provided for present study in >99 % purity. Suberoylanilide hydroxamic acid (SAHA; vorinostat), vinblastine, 3-methyladenine (3-MA), bafilomycin A1, acridine orange and monodansylcadaverine were obtained from Sigma-Aldrich Corp. (St. Louis, MO). Homoharringtonine was purchased from Santa Cruz Biotechnology (Santa Cruz, CA). Primary and secondary antibodies were from commercial sources and used according to the recommendations of the supplier. Antibodies to Cyclin B1, Cyclin D1, SQSTM1/p62, LC3B, PARP and Caspase 3 were purchased from Cell Signaling Technology, Inc. (Beverly, MA). The antibody for Actin was from Sigma-Aldrich Corp. Secondary anti- rabbit antibodies coupled to horseradish peroxidase (HRP) were from Cell Signaling Technology, Inc.

### Cell culture

Human melanoma cancer cells designated MDA-MB-435 were purchased from the American Type Culture Collection (Manassas, VA). Cells were propagated at 37 °C in 5 % CO_2_ in RPMI 1640 medium supplemented with fetal bovine serum (10 %), penicillin (100 units/mL), and streptomycin (100 μg/mL). Wild type and ATG7-deficient mouse embryonic fibroblast cells (MEF and MEF-atg7^–/–^) were kindly provided by Dr. Masaaki Komatsu (Tokyo Metropolitan Institute of Medical Science). MEFs were maintained in Dulbecco’s Modified Eagle’s Medium, supplemented with 10 % fetal bovine serum, and penicillin (100 units/mL), and streptomycin (100 μg/mL).

For the evaluation of LC3 puncta, the plasmid EGFP-LC3 was transfected into MDA-MB-435 cells with Lipofetamine 2000 Reagent from Invitrogen (Carlsbad, CA). Stable cell lines were selected using antibiotic resistant plasmids containing the gene of interest. The EGFP-LC3 plasmid was kindly provided by Dr. Wei-Pang Huang (Department of Life Science, National Taiwan University, Taipei, Taiwan). MDA-MB-435 cells stably expressing EGFP-LC3 were selected using 400 μg/mL G418 (Gemini Bio-products, West Sacramento, CA) and maintained in RPMI 1640 medium containing 200 μg/mL G418.

### Cell viability assay

Cells in log phase growth were harvested by trypsinization followed by two PBS washings to remove all traces of enzyme. Cells were seeded at 5,000 cells per well in 96-well clear, flat-bottom plates and incubated overnight. Silvestrol dissolved in DMSO was then diluted and added to the appropriate wells. The cells were incubated in the presence of silvestrol for 72 h and evaluated for viability with a commercial absorbance assay (CellTiter 96® AQueous One Solution Cell Proliferation Assay, Promega, Madison, Wisconsin) that measured viable cells. IC_50_ values were expressed in nM relative to the solvent (DMSO) control.

### Cell cycle assay

MDA-MB-435 cells were plated in 6-well plates and treated with vehicle, or silvestrol for 24 h. After treatment, the cells were collected by trypsinization, fixed in 70 % ethanol, washed in PBS, resuspended in 1 mL of PBS containing 1 mg/mL RNase and 50 μg/mL propidium iodide, incubated in the dark for 30 min at room temperature, and analyzed using an EPICS flow cytometer (Beckman-Coulter, Brea, CA). The data were analyzed using Multicycle software (Phoenix Flow Systems, San Diego, CA).

### Confocal microscopy

For detection of acidic vesicular organelles with monodansylcadaverine staining, MDA-MB-435 cells were plated 1 day before their treatment and incubated overnight. After a 24-h treatment, cells were incubated for 10 min with monodansylcadaverine (50 mM) and subsequently observed by confocal microscopy (Zeiss LSM 710, Jena, Germany). For live cell imaging detection with acridine orange, MDA-MB-435 cells were grown on MatTek 35 mm glass-bottomed culture dishes (MatTek Corp., Ashland, MA) in complete medium. Cells were exposed to silvestrol for the indicated time, then incubated with acridine orange (1 μg /mL) for 15 min and observed under a confocal microscope. For LC3 puncta imaging, MDA-MB-435 cells stably transfected with the EGFP-LC3 plasmid were grown on MatTek 35 mm glass-bottomed culture dishes, followed by silvestrol treatment for 24 h. The subcellular distribution of EGFP-LC3 was observed by confocal microscopy.

### Caspase activity assay

MDA-MB-435 cells in log-phase growth were seeded in white-walled, clear-bottomed 96-well microtiter plates and incubated overnight. The next morning, DMSO (vehicle), vinblastine or silvestrol were added to the cells to a final volume of 100 μL. After 24–48 h incubation, caspase 3/7 activity was assessed using a commercial luminescence kit according to the manufacturer’s instructions (Caspase-Glo® 3/7 Assay, Promega Corp.). The Caspase-Glo reagent, which also serves to lyse the cells, was added to each well (100 μL) and the contents were mixed gently and incubated at room temperature for 90 min. The resulting luminescence was measured using a Synergy microplate reader (BioTek Instruments, Winooski, VT).

### Morphological analysis of apoptotic cells

MDA-MB-435 cells were incubated with DMSO, 25 nM silvestrol, or 1 nM vinblastine for 24 h. The cells were then washed with PBS, fixed with 3.7 % paraformaldehyde and permeabilized with 0.1 % Triton X-100. The cells were incubated for 5 min in the dark with DAPI (250 ng/mL). The stained cells were viewed by fluorescence microscopy. The percentage of apoptotic cells was calculated as the ratio of apoptotic cells to total cells counted, and at least three fields in each well were counted.

### Flow cytometry

For the apoptosis assay, MDA-MB-435 cells were grown to confluence, and incubated with DMSO or 25 nM silvestrol for the indicated time intervals. The cells were trypsinized, washed in PBS, and centrifuged at 200× g for 5 min. Then, the pellet was resuspended in 100 μL binding buffer and added to 2 μL Annexin V-FITC and 2 μL propidium iodide (PI). After 15 min incubation at room temperature, FITC and PI fluorescence were detected using a FACScalibur flow cytometer (Becton Dickinson, San Diego, CA) and subsequently analyzed by CellQuest software (BD Biosciences, Franklin Lakes, NJ).

### qPCR analysis

Total RNA was isolated using the RNeasy kit (Qiagen, Valencia, CA) and reverse transcribed into cDNA with the high-capacity cDNA Reverse transcription kit (Applied Biosystems, Carlsbad, CA). cDNAs were plated in triplicate in 96-well plates and followed by adding TaqMan Master Mix (Applied Biosystems) and primer probe for a final volume of 25 μL. The plates were sealed, centrifuged for 1 min, and then ran by using FAM and VIC as the detector probes for each assay.

### Immunoblot analysis

Whole cell lysates were prepared from MDA-MB-435 cells treated with silvestrol at different time intervals or with inhibitors. After treatment, cells were harvested, washed twice with PBS and re-suspended in RIPA Lysis and Extraction Buffer and incubated on ice for 15 min. After centrifugation (4 °C, 15 min and 14,000 g) supernatants containing cellular proteins were collected. Protein concentration was determined by the BCA assay (Pierce, Rockford, IL). Cell lysates were adjusted for protein content and equal amounts (25 μg) separated by SDS-PAGE. Proteins were immobilized onto PVDF membranes. After saturating with 5 % (w/v) non-fat milk in TBST for 1 h at room temperature, the membranes were incubated with primary antibody overnight at 4 °C. The next day, membranes were washed in TBST (5 × 5 min) then further incubated with horseradish peroxidase-conjugated IgG secondary antibody at room temperature for 1 h followed by extensive washing with TBST (5 × 5 min). Finally, the proteins were visualized using an enhanced chemiluminescence (ECL) reagent.

### Statistical analysis

The data were analyzed by one-way analysis of variance (ANOVA) followed by a paired student t-test comparing untreated controls and treatment groups. *P*-values of 0.05 or less were considered statistically significant.

Ethics approval was not required in this study.

## Results

### Cytotoxic effect of silvestrol on human melanoma cells

Many anticancer strategies currently used in clinical oncology such as γ-irradiation, suicide gene therapy or immunotherapy, have been linked to activation of the intrinsic and/or extrinsic pathway of apoptosis in cancer cells. Silvestrol has displayed sub-nanomolar potency as a cytotoxic agent in many of the human and murine cancer cell lines in which it has been tested [6-12]. Thus, silvestrol was investigated for its ability to kill cancer cells by inducing apoptosis. To explore the molecular mechanism of silvestrol, the MDA-MB-435 human melanoma cancer cell line was treated. The IC_50_ values of silvestrol were determined using the MTS cell viability assay. As shown in Table 1, silvestrol-induced cytotoxicity was concentration-dependent when tested between the range of 100 μM and 0.01 nM. The IC_50_ value (half maximal inhibitory concentration) of silvestrol was 1.6 nM in MDA-MB-435 cells and its response to standard chemotherapy drugs is shown in Table 1. Its cytotoxicity against other cell lines was listed in Additional file 1: Table S1. Surprisingly, silvestrol appeared to be more potent than another translation inhibitor, homoharringtonine (HHT), which is approved for the treatment of chronic myeloid leukemia [27].

**Table 1:** Cytotoxicity of silvestrol on human melanoma cells. Concentration-dependent response for silvestrol-induced cytotoxicity in MDA-MB-435 cells. Melanoma cells were treated with vinblastine, silvestrol, bortezomib, homoharringtonine, and SAHA for 3 days. Cytotoxicity was determined by the MTS assay with the viability of control cells defined as 100 %. Dose-response data represent mean viability ± SE (*n* = 3 wells per treatment)

	IC_50 _(nM)
Vinblastine	1.3
Silvestrol	1.6
Bortezomib	5.7
Homoharringtonine	20
SAHA	475

### Silvestrol decreases proliferation

**Fig. 1:**
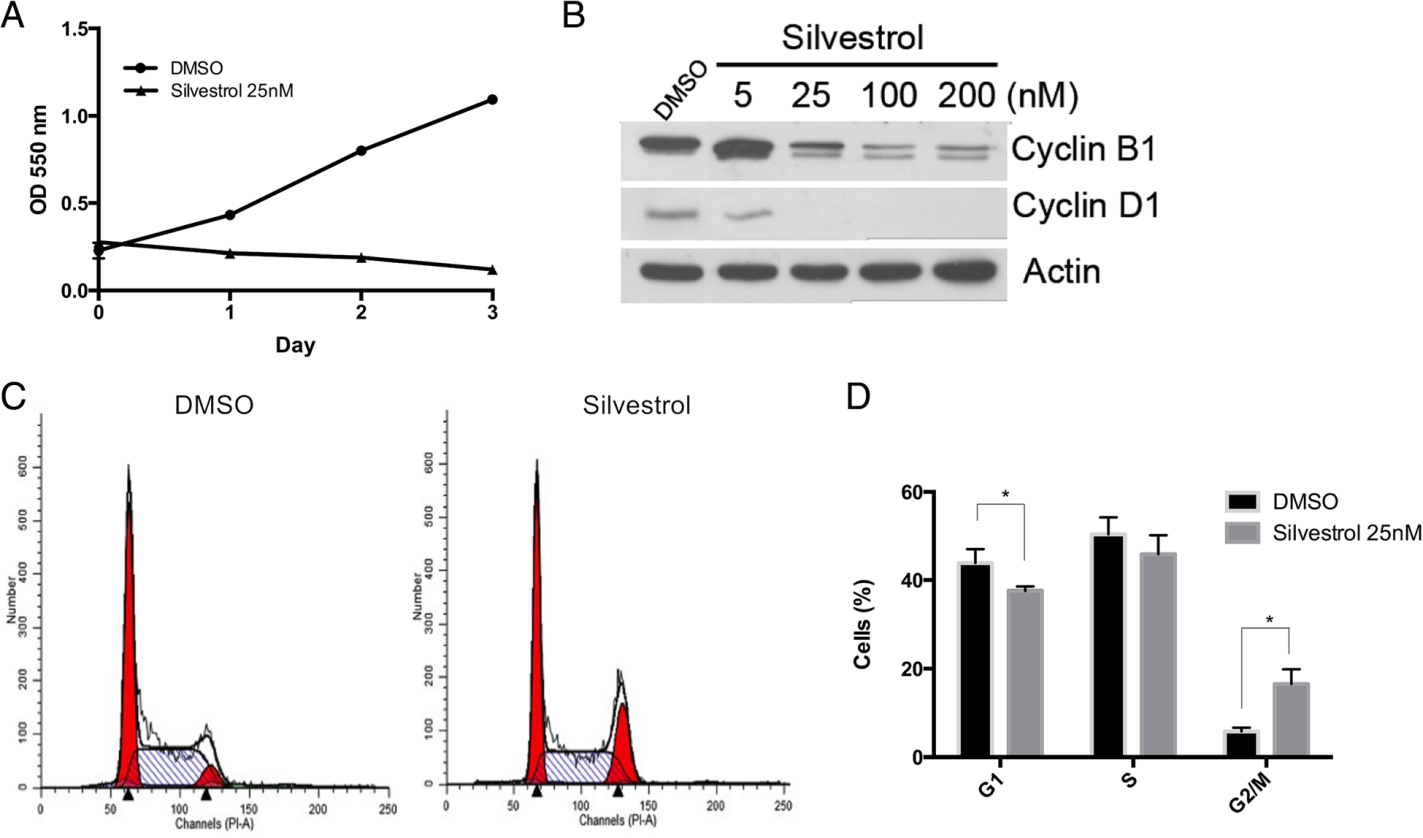
Silvestrol inhibits cell proliferation in MDA-MB-435 human melanoma cells. **a** Cells were exposed to silvestrol at 25 nM for the indicated times and evaluated for survival by the MTS assay. **b** Immunoblot analysis of cyclin B1 and cyclin D1. Cells treated with DMSO and different doses of silvestrol for 24 h and harvested. Cell lysates were resolved in SDS-PAGE and probed with specific antibodies against cyclin B1, cyclin D1 and β-actin. **c** Silvestrol inhibits cell proliferation by inducing G_2_-phase accumulation. Cells were treated with DMSO or silvestrol for 24 h prior to analysis by flow cytometry. **d** Graph displaying differences in cell cycle phases. Data represented as means ± SEM, * *p* ≤0.05

To examine whether silvestrol-induced toxicity is associated with cell cycle arrest, cells were treated with 25 nM silvestrol for 1, 2 or 3 days followed by a MTS assay. As shown in Fig. 1a, silvestrol reduced the proliferation rate of cells in a time-dependent manner. Similar results were found in the HT-29 human colon cancer cell model (Additional file 2: Figure S1). By 24 h, decreases in cyclin B1 and cyclin D1 expression were observed in silvestrol-treated cells relative to controls (Fig. 1b). Lastly, to evaluate cell cycle distribution, cells were treated with silvestrol for 24 h. The data suggested that silvestrol blocks progression through the cell cycle at the G_2_-phase (Fig. 1c and d). Silvestrol induced cell cycle arrest was also observed in HT-29 human colon cancer cells (Additional file 3: Figure S2). Taken together, these results indicate that silvestrol blocks the cell cycle at least in part by inhibiting cyclin expression.

### Silvestrol induces activation of caspase-3/7 and apoptosis

**Fig. 2:**
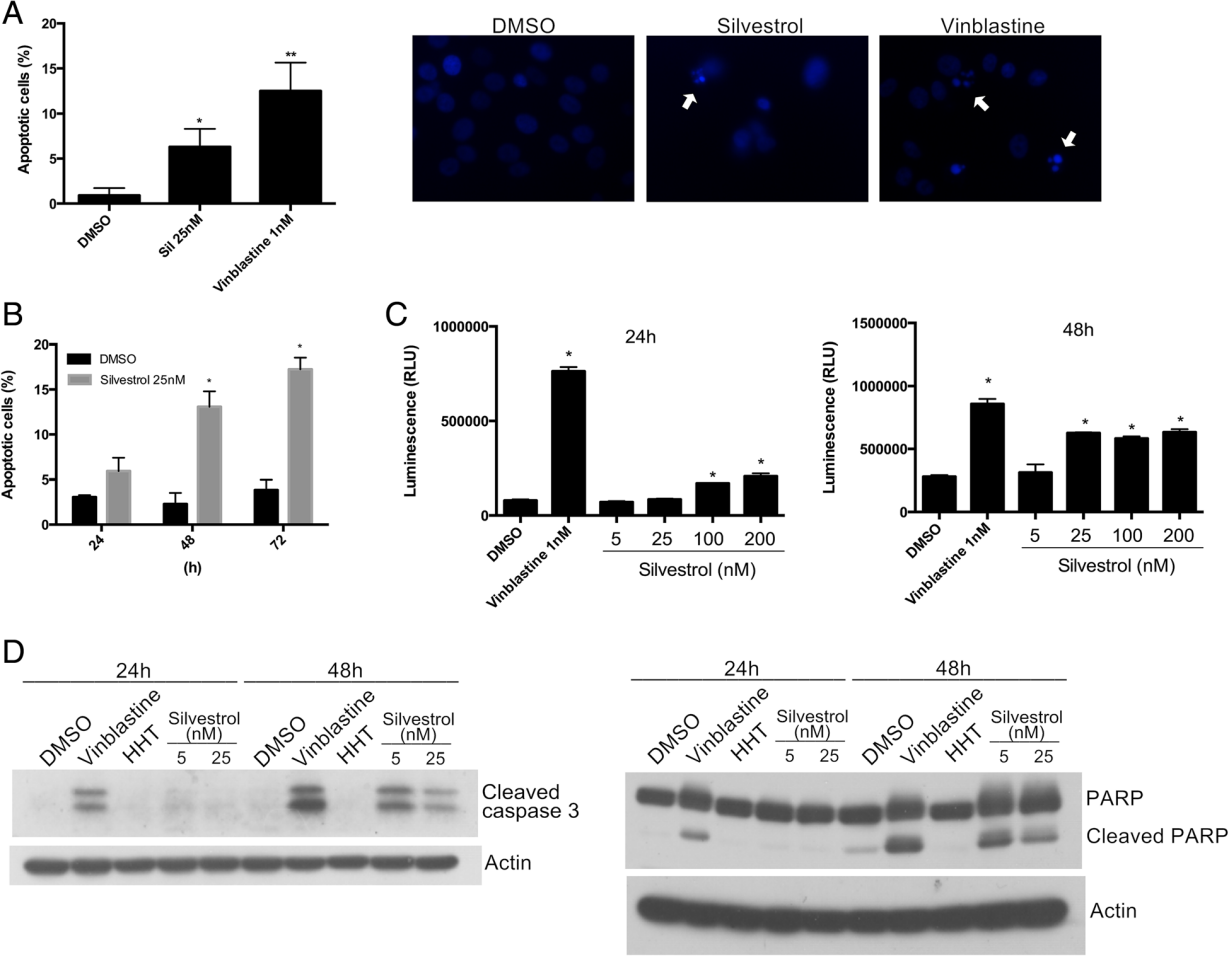
Silvestrol induces apoptosis in MDA-MB-435 cells. **a** Quantification of apoptosis was performed using DAPI staining. Apoptotic cells were identified by condensation and fragmentation of the nuclei. **b** Silvestrol induced apoptosis is time-dependent. MDA-MB-435 cells were treated with DMSO or 25 nM silvestrol for 24 to 72 h, and the Annexin V-FITC/PI double-staining analysis was performed. The early apoptosis (Annexin V-FITC positive, PI negative) and necrotic/late apoptotic (Annexin V-FITC positive, PI Positive) stages were quantified as apoptotic cells. **c** Cells in logarithmic growth were treated with DMSO, silvestrol, or vinblastine for 24 h or 48 h. Caspase 3/7 activity was assessed as production of a luminescent product. **d** Immunoblot analysis of caspase 3 and PARP. The cleavages of caspase 3 and PARP were detected in cells treated with DMSO, 1 nM vinblastine, 30 nM homoharringtonine (HHT) or silvestrol for the indicated times and harvested for protein analysis. Data presented as means ± SEM, * *p* ≤0.05

To provide some insight into the potential mechanism of silvestrol-induced cell death, the ability of silvestrol to activate apoptosis was tested. First, apoptotic cells were identified by chromatin morphology using DAPI (4',6-diamidino-2-phenylindole) staining. Silvestrol induced chromatin condensation in MDA-MB-435 cells compared to the negative control and the positive control, vinblastine (Fig. 2a). Next, flow cytometry was conducted using annexin V (AnnV) staining and propidium iodide (PI) staining to label MDA-MB-435 cells undergoing apoptosis from treatment with or without silvestrol. In the presence of silvestrol, AnnV^+^PI^+^ (late-stage apoptosis) cells significantly increased (Fig. 2b).

Caspase activation is a hallmark of the early stages of apoptotic cellular death. Within the identified major caspases, the effector, or executioner caspases are caspase-3, -6, and -7 [28]. Additionally, caspase-3/7 activation can be detected by the cleavage of a luminogenic substrate containing the sequence DEVD. Caspase-3/7 activation was detected in MDA-MB-435 cells in response to silvestrol treatment at 24 and 48 h (Fig. 2c). Similarly, the increase in caspase 3/7 activity was also detected in cells treated with vinblastine. Western blots further confirmed silvestrol induced an increase in the abundance of cleaved poly (ADP-ribose) polymerase (PARP) and cleaved caspase 3 after 48 h treatment (Fig. 2d). An increase in cleaved caspase 3 and cleaved PARP expression was observed after 48 h and was comparable to that induced by vinblastine. Next, HHT served as translation inhibitor control. Notably, cells treated HHT exhibited no increase in cleaved caspase 3 or PARP. Silvestrol is a translation inhibitor. Thus, at the protein level, the western blots showed a lower expression but a higher activity. Taken together, these findings indicate that silvestrol can induce apoptosis via activation of a caspase-3-dependent pathway in MDA-MB-435 cells. Furthermore, different translation inhibitors, such as silvestrol and HHT, do not equally induce apoptosis.

### Silvestrol induces morphological features of autophagy

**Fig. 3:**
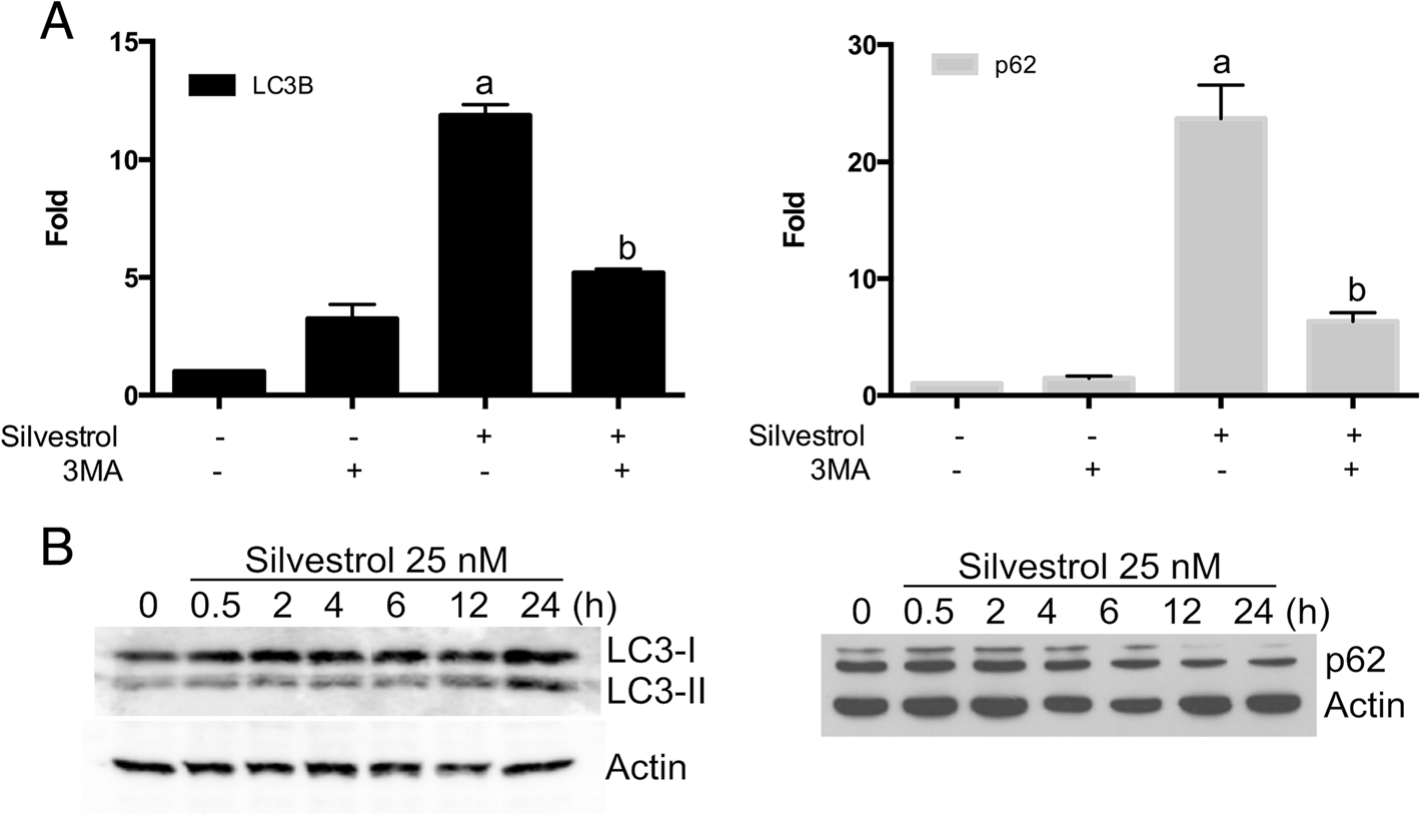
Silvestrol induces transcriptional and translational changes in MDA-MB-435 cells. **a** Cells were incubated with 3MA (10 mM), silvestrol (25 nM), or both for 24 h. mRNA expression was assessed by qPCR. **b** Silvestrol induced p62 degradation and LC3-II accumulation. MDA-MB-435 cells were treated with 25 nM silvestrol from 0-24 h and harvested for protein analysis. The data are represented as means ± SEM, * *p* ≤0.05

In order to determine additional novel mechanisms of silvestrol-mediated toxicity, a transcriptional array was performed for cancer signal transduction pathways. Preliminary data suggested that silvestrol could induce autophagy in human colon cancer cells (Additional file 4: Figure S3). Autophagy involves sequestering cytoplasmic proteins into lytic components and is characterized by the formation and promotion of acidic vesicular organelles. Therefore, to investigate silvestrol-induced toxicity further, the potential of this compound to induce autophagy was studied using biochemical and morphological criteria. In MDA-MB-435 cells, the transcriptional response to silvestrol was analyzed by using qPCR analyses. Autophagic mRNA LC3B and p62 were upregulated in the presence of silvestrol (Fig. 3a). Silvestrol’s ability to induce LC3 and p62 was blocked when combined with the autophagy inhibitor, 3-methyladenine (3MA) (Fig. 3a). In HT-29 colon cancer cells, silvestrol-induced autophagy related gene expression was also inhibited by 3MA (Additional file 5: Figure S4). Consequently, these results were consistent with the hypothesis that silvestrol exposure can induce gene transcription associated with autophagy. Next, western blots were performed under the same conditions to confirm changes in protein expression consistent with autophagy. The expression of endogenous LC3 was analyzed in lysates derived from MDA-MB-435 cells that had been treated with silvestrol for up to 24 h. Silvestrol treatment induced early autophagy in the MDA-MB-435 cells, which was characterized by cleavage of LC3 and a decline in the p62 levels relative to control (Fig. 3b). These results imply silvestrol is inducing the early stages of autophagy in MDA-MB-435 cells.

### Silvestrol induces autophagosome accumulation

**Fig. 4:**
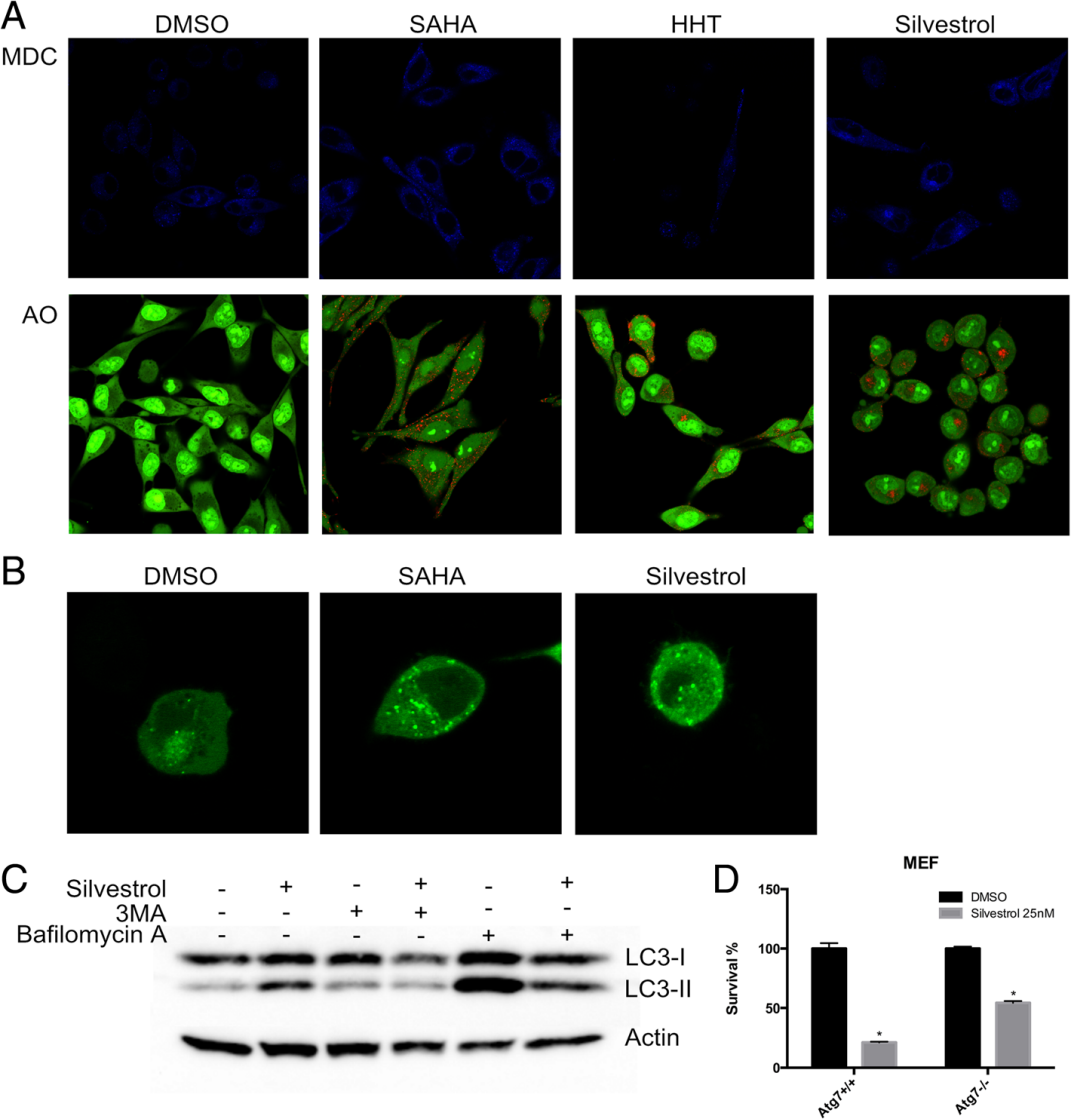
Silvestrol treatment induces autophagy in MDA-MB-435 cells. **a** Cells were treated with either DMSO, 5 μM SAHA, 30 nM homoharringtonine (HHT), or 25 nM silvestrol for 24 h and subsequently stained with monodansylcadaverine (MDC) or acridine orange (AO). Both silvestrol and SAHA promoted vacuole formation (indicated by blue staining in MDC and red staining in AO). Images are representative of a pattern of staining observed in at least three independent experiments. **b** Silvestrol induces autophagy by mediating EGFP-LC3 translocation. Representative pictures of cells treated with DMSO, 5 μM SAHA or 25 nM silvestrol for 24 h. **c** Cells were treated with 25 nM silvestrol in the presence or absence of 3MA (10 mM), bafilomycin A1 (50 nM) and then harvested for protein analysis. Cell lysates were resolved in SDS-PAGE and probed with specific antibodies against LC3 and Actin. **d** Wild-type (WT) or Atg7^-/-^ MEFs were treated with 25 nM silvestrol for 72 h. Cytotoxicity was determined by MTS assay with the viability of control cells defined as 100 %. The data are represented as means ± SEM, * *p* ≤0.05

In order to quantify autophagolysosome-containing cells, treated cells were stained with monodansylcadaverine (MDC). Vital staining of silvestrol-treated MDA-MB-435 cells with MDC, a specific autophagolysosome marker, revealed increased accumulation of the dye relative to control cells (Fig. 4a). The increase in autophagolysosome was comparable to that observed with HHT and SAHA, which is known to induce autophagy [29]. To quantify the development of the acidic vesicular organelles, silvestrol-treated cells were stained with acridine orange. Representative photomicrographs of control, SAHA-treated, HHT-treated, and silvestrol-treated cells are shown. Both silvestrol and SAHA enhanced autophagosome formation in MDA-MB-435 cells (Fig. 4a).

To better understand the role of silvestrol in autophagosome formation, LC3 protein location was evaluated. LC3, a homolog of yeast Atg8, was used as an autophagy marker. LC3 is specifically localized to autophagic structures, including the autophagosome and its precursor structures, the phagophore and the autolysosome. Under normal nutritional conditions, LC3 protein is distributed diffusely in the cytoplasm. Upon induction of autophagy (e.g., by starvation), autophagosomes are formed, which is an important early step of autophagy induction, while LC3 gets redistributed to a vacuolar pattern. The EGFP-LC3 fusion protein has a similar distribution pattern to endogenous LC3 and can be used as an alternative marker of autophagy induction since it appears as cytoplasmic puncta under fluorescence microscopy. Therefore, the appearance of multiple LC3-positive puncta suggests the induction of autophagy. MDA-MB-435 EGFP-LC3 cells were used to study the cell response to silvestrol. When comparing with control cells, cells treated with silvestrol or SAHA displayed multiple EGFP-LC3 puncta, representing autophagic vacuoles that were formed in the cytoplasm (Fig. 4b).

An increased number of autophagosomes could result from either increased formation or decreased degradation. 3MA, a PI3K inhibitor, blocks autophagy induction. Bafilomycin A1 is a potent inhibitor for vacuolar-type H^+^-ATPase that is required for fusion of the autophagosome with the lysosome. Therefore, 3MA and bafilomycin A1 were employed to assess LC3-II accumulation as a marker of autophagosome formation in MDA-MB-435 cells. As illustrated in Fig. 4c, silvestrol-induced LC3-II accumulation was attenuated by 3MA. In contrast, silvestrol could still induce LC3-II accumulation in the presence of bafilomycin A1, suggesting that the increase of LC3-II was not due to the blockage of autophagic degradation.

In mammalian cells, Atg7 is essential for the autophagy conjugation system, the formation of autophagosomes, and degradation of proteins and organelles. To evaluate the relationship between autophagy and silvestrol-induced cell death, silvestrol-induced cytotoxicity in wild-type and Atg7-null mouse embryonic fibroblasts (MEFs) were compared. The cytotoxicity induced by silvestrol was more sensitive in wild-type compared to Atg7^-/-^ MEF cells (Fig. 4d), suggesting autophagy was involved in silvestrol-induced cell death. Taken together, these findings suggest that silvestrol upregulates gene transcription and triggers the protein translation of the early autophagy pathway components as well as caspase-mediated apoptosis.

## Discussion

Silvestrol is a plant-derived natural product that represents a promising lead structure in anti-cancer drug discovery. In this study, the cytotoxic potential of silvestrol against human melanoma cells was investigated. Silvestrol dramatically reduced the viability of MDA-MB-435 cells. Additionally, decreases in cyclin B1 and cyclin D1 expression were observed in silvestrol-treated cells, with blockage of the cell cycle at the G_2_-phase. Silvestrol also induced apoptotic features such as nuclear chromatin condensation and caspase-3 activation. Silvestrol induced early autophagosome accumulation, such as LC3-II accumulation and time-dependent p62 degradation. The upstream inhibitor, 3MA, but not the downstream inhibitor, bafilomycin A, blocked autophagy processes induced by silvestrol. Taken together, these studies have provided insight into the potential of silvestrol to induce cell death in melanoma.

Previous data have demonstrated that silvestrol can induce cell cycle arrest and apoptosis [30, 31]. However, little is known about the molecular mechanism(s) mediating these effects. Cell death can occur through multiple pathways, and the induction of multiple mechanisms of cell death might be useful in cancer therapy. Inhibition of protein synthesis leading to autophagy and apoptosis is a promising new strategy for anticancer therapy. For instance, the histone deacetylase (HDAC) inhibitor, SAHA, is a strong autophagy inducer and also initiates caspase-dependent apoptosis [32, 33]. In contrast, sanguilutine is another natural product that has been documented to induce autophagy, but it does not induce caspase-dependent cell death in human A375 melanoma [34]. These studies support that silvestrol induces both early autophagy and caspase-mediated apoptosis in human melanoma cells. These activities are different when compared to homoharringtonine, another protein synthesis inhibitor, highlighting the different mode of translation inhibition. Driven by the need for new anticancer targets, the exploration of small molecules that regulate cell death through several mechanisms may provide valuable cancer chemotherapeutic agents.

There is accumulating evidence that modulation of protein translation with depletion of short half-life survival factors can enhance therapeutic responses [31]. The effect of silvestrol on cyclin B and cyclin D expression is consistent with these reports. Further analysis of selectively translated mRNAs modulated by silvestrol may be useful to understand specific pathways involved in cancer progression.

There are strong correlations between defects in autophagy regulation or execution and cancer development [35]. This may be due to the fact that autophagy deficiency results in increased DNA damage and gene amplification, decreased cellular differentiation and protein catabolism, especially during stress. In addition, evidence is accruing in the literature that suggests that chemotherapeutic agents can induce autophagic cell death in apoptosis deficient cancer cells while autophagy might potentiate some anticancer drugs against cancer [29, 32, 36–38]. In contrast, other studies suggest that genetic or pharmacological inhibition of autophagy enhances efficacy of cancer chemotherapeutic agents [39, 40]. Suppression of the early stage of autophagy by Atg7 knockout reduced silvestrol-induced cytotoxicity indicating that autophagy assists in some of the silvestrol-induced cell death. Combination therapies with silvestrol and other chemotherapeutic agents are under active investigation for melanoma and other cell lines. Although silvestrol and HHT are both as translation inhibitors, HHT can induce autophagy but has a limited ability to drive the apoptotic pathway suggesting that the mechanism of translational inhibition impacts autophagy and its relationship to apoptosis. Cycloheximide, another translational inhibitor demonstrated autophagy inhibition [41]. Subtle differences in the binding site of the specific translational inhibitor may underlie changes in autophagy.

The functional relationship between the two self-destructive processes, autophagy and apoptosis, is complex and under-studied. In general, it appears that similar stimuli can induce either apoptosis or autophagy in a mutually exclusive manner [20]. Generally, autophagy represents a stress adaptation that avoids cell death, but in several scenarios, autophagy can also lead to autophagic cell death [42–45]. For example, in previous reports inhibition of the early steps of autophagy reduced the activation of caspase 8-mediated apoptosis, while inhibition of the late steps of autophagy increased caspase-dependent cell death [46]. A recent study indicates that p62 might act as a key factor that influences autophagy to induce cell death or survival [35, 47]. It has been shown p62 silencing induced autophagy activation and caused cell death. In fact, the protein p62 can interact with TRAF6, which is a lysine 63 (K63) E3 ubiquitin ligase that promotes TRAF6 oligomerization, activation of NFκB, and cell survival during tumorigenesis [35, 48]. Thus, the elimination of p62 suppresses tumorigenesis. Based on the current study, silvestrol similarly leads to the degradation of p62 and cancer cell death.

## Conclusion

In summary, silvestrol is an unusual rocaglate derivative with a dioxanyl ring that potently inhibits cell growth and induces cell death in human melanoma cells through induction of early autophagy and caspase-mediated apoptosis. These findings provide insight into the mechanisms of cell death and signaling from silvestrol in melanoma. Understanding the interplay between autophagy and apoptosis could potentially inform the development of future chemotherapy agents and improve combination therapies that stimulate these pathways. Future studies on silvestrol could focus on structure/ligand based drug design in concert with structural biology, synthetic chemistry, biochemical analysis and pharmacokinetics [49]. Based on the cytotoxic potential of silvestrol, this may provide a novel and promising strategy to improve the anticancer treatment.

## Additional file

Additional file 1: Table S1. IC_50_ of silvestrol in human cancer cell lines. (DOC 27 kb) Additional file 2: Figure S1. Silvestrol inhibits cell proliferation in HT-29 human colon cancer cells. Cells were exposed to silvestrol at 25 nM for the indicated times and evaluated for survival by the MTS assay. (PNG 316 kb) Additional file 3: Figure S2. Silvestrol inhibits cell proliferation by inducing G_2_-phase accumulation in HT-29 cells. Cells were treated with DMSO or silvestrol for 24 h prior to analysis by flow cytometry. (PNG 313 kb) Additional file 4: Figure S3. Expression of autophagy related genes induced by silvestrol treatment. HT-29 cells were treated with 25 nM silvestrol for 16 h and mRNA of cells were assessed. Equivalent mRNA loading was assessed by internal control. (PNG 601 kb) Additional file 5: Figure S4. Silvestrol induces transcriptional changes in HT-29 cells. Cells were incubated with 3MA (10 mM), silvestrol (25 nM), or both for 24 h. mRNA expression was assessed by qPCR. The data are represented as means ± SEM, * *p* ≤0.05. (PNG 567 kb)

## Abbreviations

3MA: 3-methyladenine
AO: acridine orange
DAPI: 4',6-diamidino-2-phenylindole
EGFR: epidermal growth factor receptor
HHT: homoharringtonine
LC3: light chain 3
MDC: monodansylcadaverine
NExT: National Cancer Institute Experimental Therapeutics
PARP: poly (ADP-ribose) polymerase
RTK: receptor tyrosine kinases
SAHA: suberoylanilide hydroxamic acid
