# Supplementary material for: Silvestrol induces early autophagy and apoptosis in human melanoma cells
Source: BMC Cancer. 2016 Jan 13;16:17. doi: 10.1186/s12885-015-1988-0 (PMC4712514; doi:10.1186/s12885-015-1988-0)
Supplement: Additional file 1: Table S1. — IC50 of silvestrol in human cancer cell lines. (DOC 27 kb) [file 12885_2015_1988_MOESM1_ESM.doc]

Table S1. IC_50_ of silvestrol in human cancer cell lines

|  | Silvestrol |
| --- | --- |
| MDA-MB-435 | 1.60 nM |
| MDA-MB-231 | 3.18 nM |
| OVCAR3 | 0.47 nM |
| HT-29 | 7.09 nM |
