# Supplementary figures and images for: Silvestrol induces early autophagy and apoptosis in human melanoma cells
Source: BMC Cancer. 2016 Jan 13;16:17. doi: 10.1186/s12885-015-1988-0 (PMC4712514; doi:10.1186/s12885-015-1988-0)

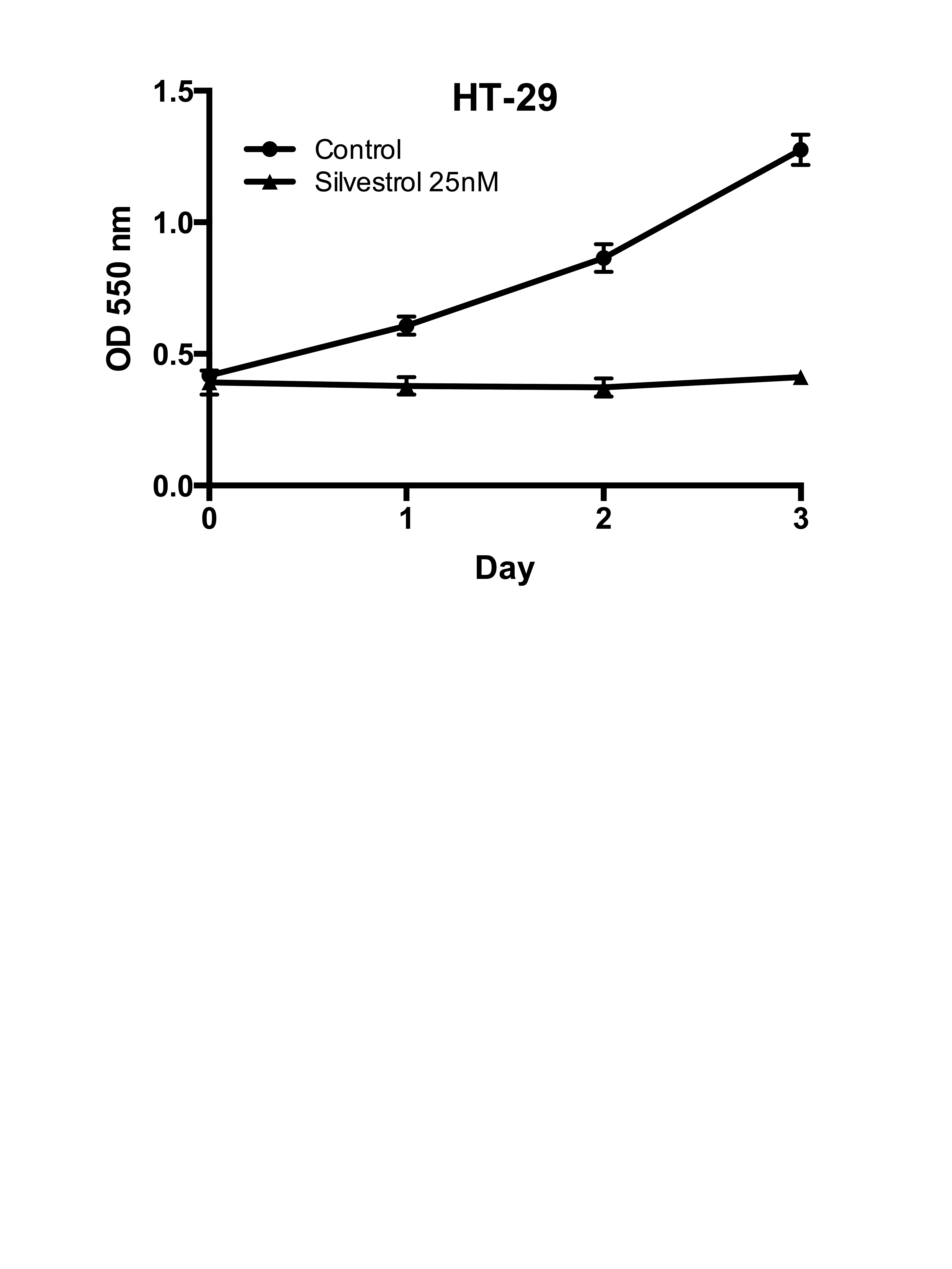

Supplement: Additional file 2: Figure S1. — Silvestrol inhibits cell proliferation in HT-29 human colon cancer cells. Cells were exposed to silvestrol at 25 nM for the indicated times and evaluated for survival by the MTS assay. (PNG 316 kb) [file 12885_2015_1988_MOESM2_ESM.png]

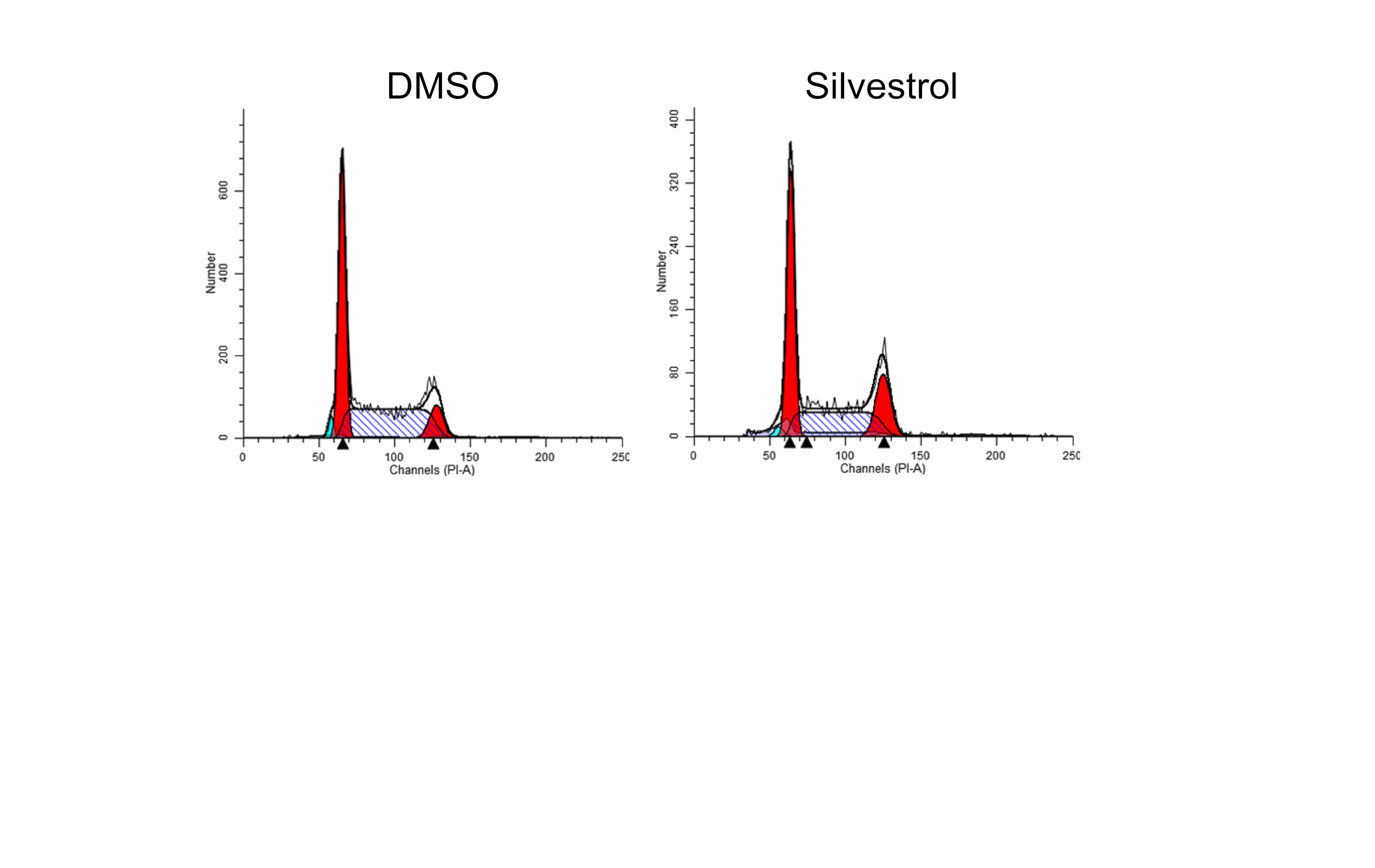

Supplement: Additional file 3: Figure S2. — Silvestrol inhibits cell proliferation by inducing G2-phase accumulation in HT-29 cells. Cells were treated with DMSO or silvestrol for 24 h prior to analysis by flow cytometry. (PNG 313 kb) [file 12885_2015_1988_MOESM3_ESM.png]

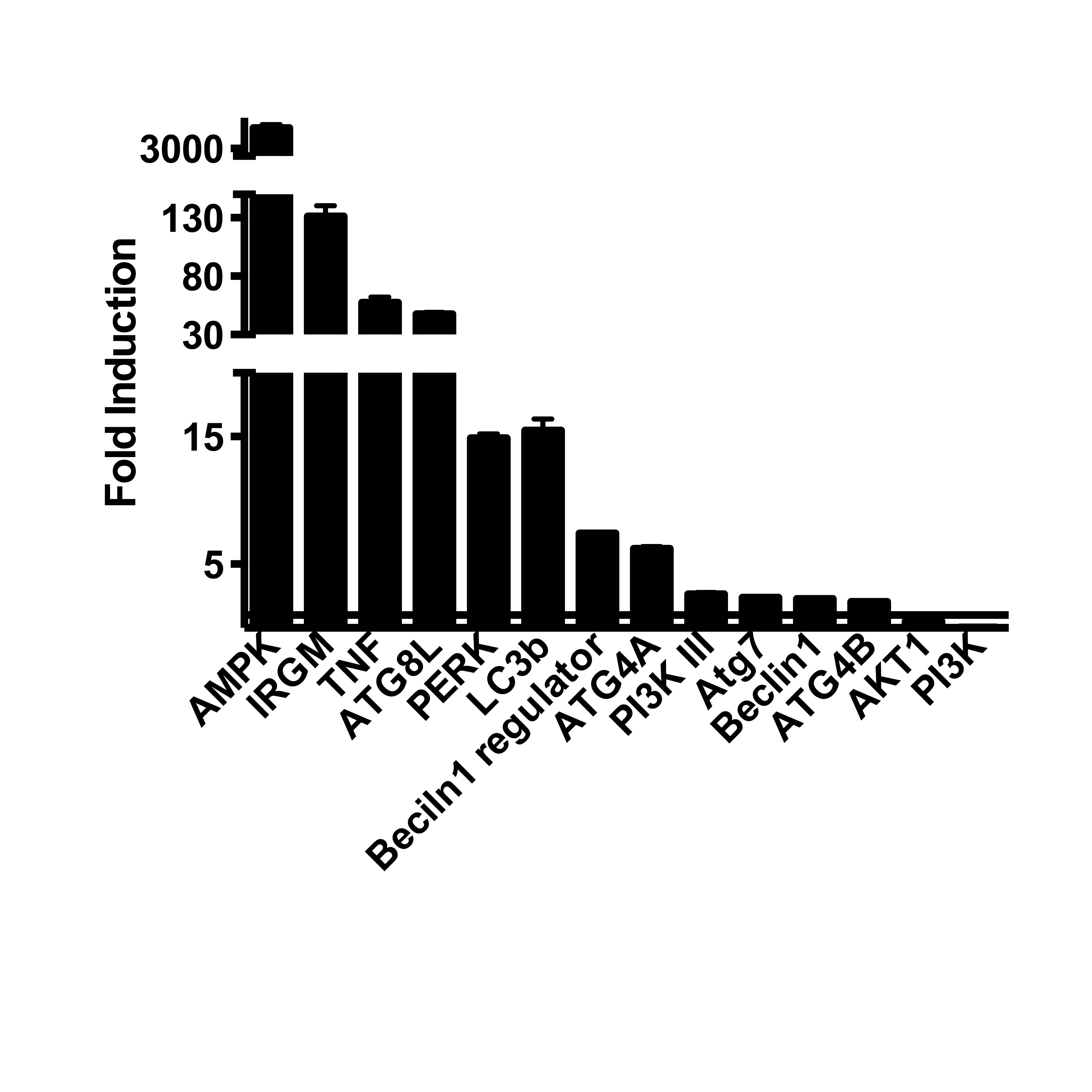

Supplement: Additional file 4: Figure S3. — Expression of autophagy related genes induced by silvestrol treatment. HT-29 cells were treated with 25 nM silvestrol for 16 h and mRNA of cells were assessed. Equivalent mRNA loading was assessed by internal control. (PNG 601 kb) [file 12885_2015_1988_MOESM4_ESM.png]

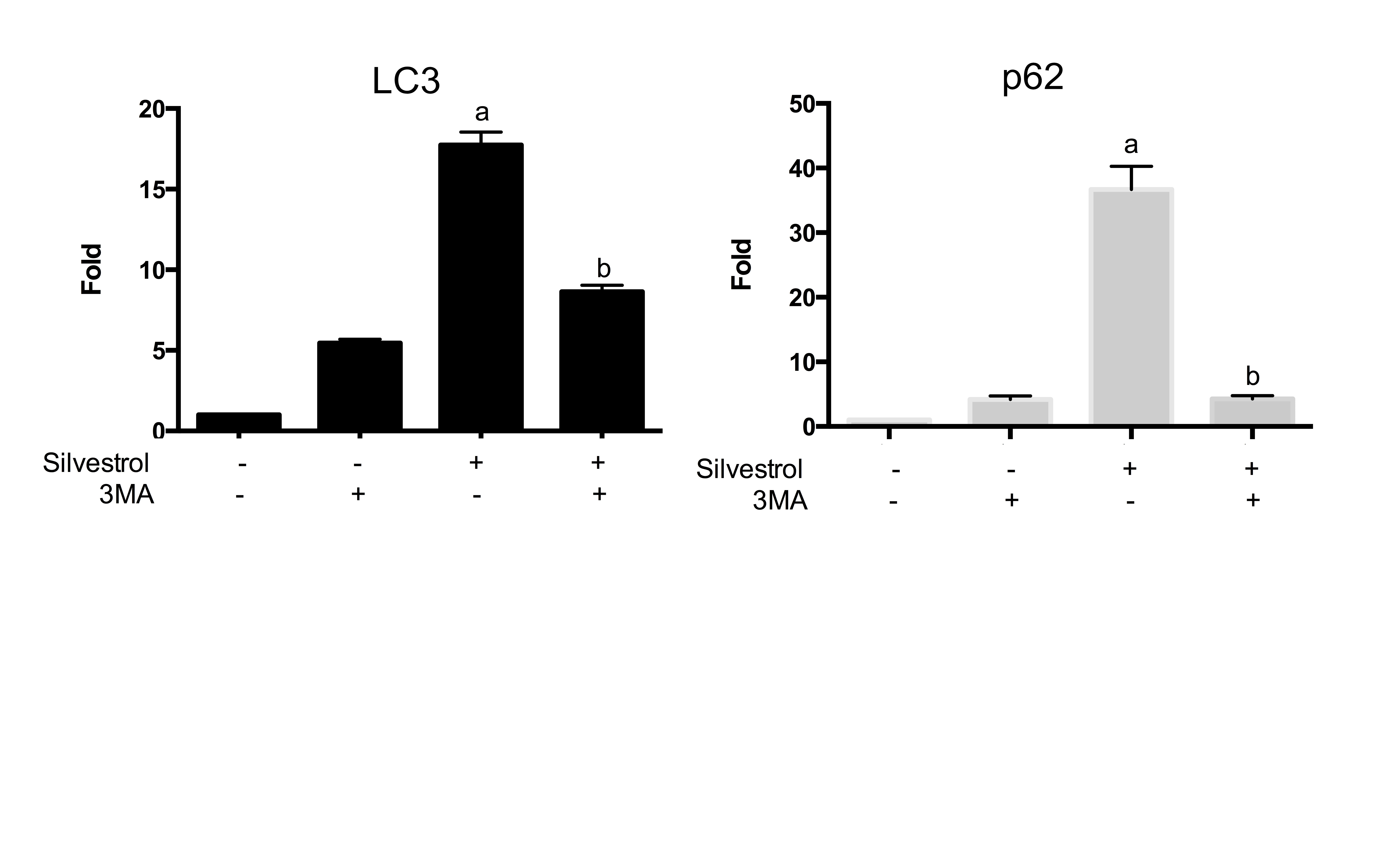

Supplement: Additional file 5: Figure S4. — Silvestrol induces transcriptional changes in HT-29 cells. Cells were incubated with 3MA (10 mM), silvestrol (25 nM), or both for 24 h. mRNA expression was assessed by qPCR. The data are represented as means ± SEM, * p ≤0.05. (PNG 567 kb) [file 12885_2015_1988_MOESM5_ESM.png]
